# Systems level expression correlation of Ras GTPase regulators

**DOI:** 10.1186/s12964-018-0256-8

**Published:** 2018-08-15

**Authors:** E. Besray Unal, Christina Kiel, Hannah Benisty, Andrew Campbell, Karen Pickering, Nils Blüthgen, Owen J. Sansom, Luis Serrano

**Affiliations:** 1 Institute of Pathology, Charité - Universitätsmedizin Berlin, 10117 Berlin, Germany; 2 Integrative Research Institute Life Sciences, Humboldt Universität Berlin, 10115 Berlin, Germany; 3 Centre for Genomic Regulation (CRG), Systems Biology Programme. The Barcelona Institute of Science and Technology, Dr. Aiguader 88, Barcelona, 08003 Spain; 4 Present address: Systems Biology Ireland & Charles Institute of Dermatology & School of Medicine, University College Dublin, Belfield, Dublin 4, Ireland; 5 Cancer Research UK Beatson Institute, Garscube Estate, Switchback Road, Glasgow, G61 1BD UK; 6 Universitat Pompeu Fabra (UPF), 08003 Barcelona, Spain; 7 Institució Catalana de Recerca i Estudis Avançats (ICREA), Pg. Lluís Companys 23, 08010 Barcelona, Spain

**Keywords:** Ras small GTPases, Tissue expression, Gene expression network, GTPase activating proteins, Guanine nucleotide exchange factors

## Abstract

**Background:**

Proteins of the ubiquitously expressed core proteome are quantitatively correlated across multiple eukaryotic species. In addition, it was found that many protein paralogues exhibit expression anticorrelation, suggesting that the total level of protein with a given functionality must be kept constant.

**Methods:**

We performed Spearman’s rank correlation analyses of gene expression levels for the RAS GTPase subfamily and their regulatory GEF and GAP proteins across tissues and across individuals for each tissue. A large set of published data for normal tissues from a wide range of species, human cancer tissues and human cell lines was analysed.

**Results:**

We show that although the multidomain regulatory proteins of Ras GTPases exhibit considerable tissue and individual gene expression variability, their total amounts are balanced in normal tissues. In a given tissue, the sum of activating (GEFs) and deactivating (GAPs) domains of Ras GTPases can vary considerably, but each person has balanced GEF and GAP levels. This balance is impaired in cell lines and in cancer tissues for some individuals.

**Conclusions:**

Our results are relevant for critical considerations of knock out experiments, where functionally related homologs may compensate for the down regulation of a protein.

**Electronic supplementary material:**

The online version of this article (10.1186/s12964-018-0256-8) contains supplementary material, which is available to authorized users.

## Background

The systematic profiling of gene and protein expression levels in different tissues and cell types has enabled the definition of common and unique components and the functional characterisation of tissues and organs (‘What makes a cell type?’) [1–4]. Expression and clustering analysis revealed that core conserved genes and proteins are expressed at similar levels in different eukaryotes [5] and across various mammalian and non-mammalian vertebrate tissues [6, 7]. However, it is unclear if there is a quantitative expression association for groups of functionally related genes and family members (homologs) that do not belong to the evolutionary conserved ubiquitous expressed core genes and proteins. Recently it has been shown that functional divergence of paralogs is fast, promoting tissue specificity [8]; in this, orthologues show a high conservation in tissue-specificity, whilst paralogs show less conservation [9]. We have shown previously that paralogous gene pairs that are less similar in sequence homology and domain composition showed expression anticorrelation profiles during the calcium-induced differentiation of primary human keratinocytes [10]. Anticorrelation of paralog gene expression was also observed in different brain regions [11]. This suggests specialized time- and space-dependent roles of different paralogs and, more importantly, the need to keep the same level of common functionalities carried out by paralogs. We could ask if the same is true for antagonistic activities regulating essential cell processes. An interesting question is if the same could happen for antagonistic activities regulating essential cell functions, such as those mediated by the family of small GTPase proteins.

**Fig. 1:**
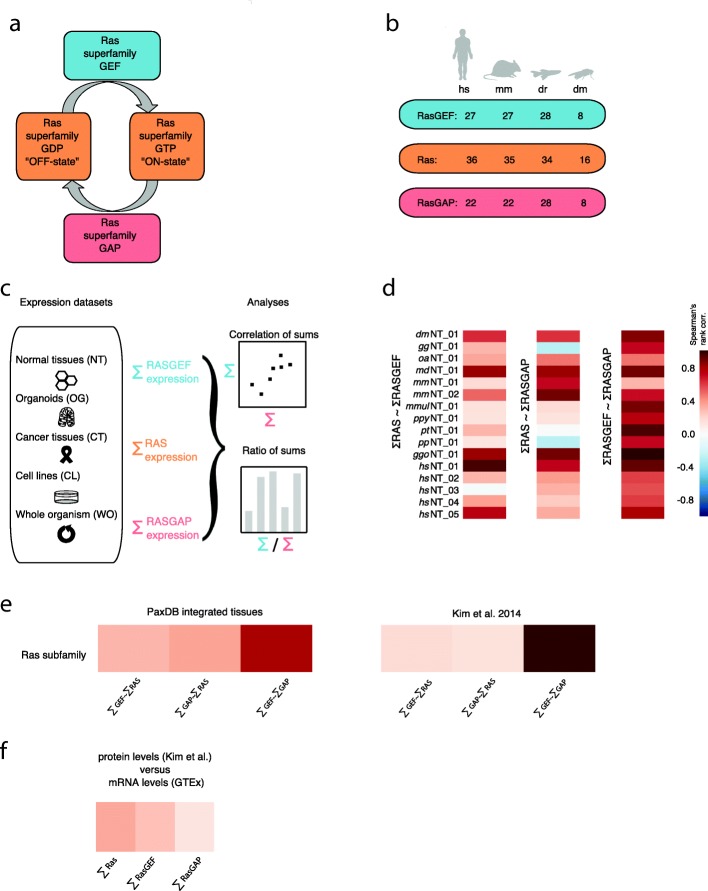
The Ras subfamily and their regulators, overview of analyses performed, and Spearman’s rank correlations. **a** Schematic diagram of the Ras superfamily of small GTPases that cycle between a GDP-bound “OFF-state” and a GTP-bound “ON-state”. This cycling is regulated by GEF and GAP proteins. **b** Number of Ras subfamily and regulators genes in four species (*Homo sapiens*, *hs*; *Mus musculus*, *mm*; *dr*, *Danio rerio*, *dr*; *Drosophila melanogaster*, *dm*). **c** Schematic figure of protein and gene expression datasets used, and main data analyses performed in this study. **d** Spearman’s rank correlation values of the ∑RAS vs ∑GEF, ∑RAS vs ∑GAP, and the ∑GEF vs ∑GAP displayed as a heat map (normal tissue datasets *dm*NT_01, *gg*NT_01, *oa*NT, *md*NT_01, *mm*NT_01, *mm*NT_02, *mmu*NT_01, *ppy*NT_01, *pt*NT_01, *pp*NT_01, *ggo*NT_01, *hs*NT_01, *hs*NT_02, *hs*NT_03, *hs*NT_04, *hs*NT_05). **e** Heatmap representation of Spearman’s rank correlations of protein levels for GEFs with RASs, GAPs with RASs, and GEFs with GAPs across human normal tissues. While correlations between the GEF with the RAS and the GAP with the RAS were generally poorer, higher correlations between the GEF with the GAP were observed independently in both proteomic datasets. **f** Heatmap representation of Spearman’s rank correlations of protein and RNA levels (*hs*NT_03). Parts of the pictures were drawn using Inkscape

To answer this question about a family-wide expression balance, we choose the Ras p21 subfamily of small GTPases [12] (associated with cancer [13]), together with their regulators, which direct diverse cellular processes by cycling between GTP-bound active and GDP-bound inactive conformations (Fig. 1a) [13, 14]. Cycling between the ‘OFF states’ (GDP-bound) and the ‘ON-states’ (GTP-bound) of Ras proteins is catalysed GTP exchange factors domains (RasGEFs) and GTP activating domains (RasGAP). The GEF and GAP regulators are multidomain proteins of diverse composition (e.g. SH2, SH3, PH, RBD domains) in addition to their catalytic GEF or GAP domains (Additional file 1: Figure S1). Because each RAS subfamily has GEFs and GAPs with unique catalytic domains of 3D structural and sequence similarity, this makes them in principle specific for each respective subfamily [15–17].

Here, we have analysed the gene and protein expression levels for the RAS GTPase subfamily and their regulatory GEF and GAP proteins using a large set of published data for normal tissues from a wide range of species, human cancer tissues and human cell lines. We show that the sum of all GEF family members (∑RASGEF) generally correlates with the sum of all GAP family members (∑RASGAP) in physiological normal tissues, although the total sums for GEFs and GAPs could change significantly between individuals. Thus, there is a balance in gene and protein expression levels of RAS regulators for normal tissues. These ∑RASGEF-∑RASGAP correlations are lost or have a different slope in transformed cell lines and in some individual cancer tissues. This is caused by a higher sum of GAP levels in cancer tissues of some individuals. Our work shows that to understand the functionality of cell processes it is important to consider all members of a protein family. Individual proteins levels can be different from tissue to tissue, but the sum of homologs is constant, possibly to ensure adequate functional balance.

## Methods

### Transcriptomics data sources

We collected the following published datasets: (*hs*NT_01) RNA-Seq gene expression dataset associated to *H. sapiens* 11 normal tissues [18] (*hs*NT_02) RNA-Seq gene expression dataset associated to *H. sapiens* 32 normal tissues (https://www.ebi.ac.uk/arrayexpress/experiments/E-MTAB-2836/); (*hs*NT_03) RNA-Seq gene expression dataset associated to *H. sapiens* 53 normal tissues [19, 20] (GTEx); (*hs*NT_04) RNA-Seq gene expression dataset associated to *H. sapiens* 18 normal tissues (TCGA Research Network, http://cancergenome.nih.gov/); (*hs*NT_05) RNA-Seq gene expression dataset associated to *H. sapiens* 8 tissues [21]; (*ggo*NT_01) RNA-Seq gene expression dataset associated to *G. gorilla* 6 tissues [21]; (*pt*NT_01) RNA-Seq gene expression dataset associated to *P. troglodytes* 6 tissues [21]; (*pp*NT_01) RNA-Seq gene expression dataset associated to *P. paniscus* 6 tissues [21]; (*ppy*NT_01) RNA-Seq gene expression dataset associated to *P. pygmaeus* 5 tissues [21]; (*mmul*NT_01) RNA-Seq gene expression dataset associated to *M. mulatta* 7 tissues [21]; (*mm*NT_01) RNA-Seq gene expression dataset associated to *M. musculus* 6 tissues [21]; (*md*NT_01) RNA-Seq gene expression dataset associated to *M. domestica* 6 tissues [21]; (*oa*NT_01) RNA-Seq gene expression dataset associated to *O. anatinus* 6 tissues [21]; (ggNT_01) RNA-Seq gene expression dataset associated to *G. gallus* 6 tissues [21]; (*mm*NT_02) microarray gene expression dataset associated to *M. musculus* 45 normal tissues [22]; (*dm*NT_01) microarray gene expression dataset associated to *D. melanogaster* 25 normal tissues [23]; (*hs*CT_01) RNA-Seq gene expression dataset associated to *H. sapiens* 18 cancer tissues (TCGA Research Network http://cancergenome.nih.gov/); (*hs*CT_02) microarray gene expression dataset associated to *H. sapiens* 31 cancer tissues [24] (COSMIC sanger cell line project); (*hs*CT_03) microarray gene expression dataset associated to *H. sapiens* 163 cancer tissues (Expo database, http://www.intgen.org/research-services/biobanking-experience/expo/); (*hs*CL_01) RNA-Seq gene expression dataset associated to *H. sapiens* 56 cell lines (Human Protein Atlas, http://www.ncbi.nlm.nih.gov/bioproject/PRJNA183192); (hsCTL_02) microarray gene expression dataset associated to *H. sapiens* cell lines from 24 cancer tissues (Cancer Cell Line Encyclopedia [25]). RPKM (Reads Per Kilobase per Million mapped reads) or TPM (Transcripts Per Kilobase Million) values were used for RNA-Seq data, while total RNA levels from microarray datasets were utilized. We have always considered the most expressed isoform for each gene.

### Proteomics data sources

The PaxDB, a mass spectrometry-based proteomic meta-resource across organisms was used to extract protein information [26]. The average spectral counting value derived from all experiments (in different cell lines and tissues) corresponds to an approximate protein abundance estimate for the respective organism (ppm values for ‘whole organisms’ and integrated tissues’ in PaxDb). To retrieve protein levels for RAS, GEF, and GAP members in different organisms and under different conditions (e.g. cell lines, normal tissues), we first analyzed the PaxDB (Additional file 2). We found a good coverage of expression information using the ‘whole organism’ expression score, which is an average spectral counting value derived from all mass spectrometry experiments from different cell lines and tissues. Similarly, we retrieved integrated expression information for 20 tissues from the PaxDB and for 20 tissues from a recent deep proteomic study [3]. However, the complete quantification of lower abundance proteins and of isoforms and family members (that often contain only a few unique or no unique tryptic peptides) by mass spectrometry in tissues is not feasible [27].

### Selection of Ras, RasGEF, and RasGAP family members

Members of the Ras family were selected based on the presence of a predicted Ras domain using either the SMART (accession number: SM00173; http://smart.embl-heidelberg.de/) or the Pfam database (accession number: PF00071; https://pfam.xfam.org/) (Table 1, Table 2, Additional file 3). All 36 Ras family members were also present in a detailed bioinformatics evolutionary tree analysis of the whole RAS superfamily [12]. Members of the RasGEF family were selected based on the presence of a predicted Ras domain using either the SMART (accession number: SM00147; http://smart.embl-heidelberg.de/) or the Pfam database (accession number: PF00617; https://pfam.xfam.org/). Members of the RasGAP/[RapGAP] family were selected based on the presence of a predicted RasGAP domain using either the SMART (accession number: SM00323; http://smart.embl-heidelberg.de/) or the Pfam database (accession numbers RasGAP: PF00616 and RapGAP: PF02145; https://pfam.xfam.org/). NF2 was excluded from the list of RasGAPs as despite its name, NF2 is not related to NF1 and does not display GAP activity against any Ras GTPase [15].

**Table 1:** Selection of Ras superfamily members. Selection of family members based on domain predictions using the SMART or Pfam databases, or based on Rojas et al., 2012

	Gene name	RAS domain in SMART	RAS domain in Pfam	Rojas et al. 2012
Ras family	DIRAS1	YES	YES	YES
	DIRAS2	YES	YES	YES
	DIRAS3	NO	YES	YES
	ERAS	YES	YES	YES
	GEM	NO	YES	YES
	HRAS	YES	YES	YES
	KRAS	YES	YES	YES
	MRAS	YES	YES	YES
	NKIRAS1	NO	YES	YES
	NKIRAS2	NO	YES	YES
	NRAS	YES	YES	YES
	RALA	YES	YES	YES
	RALB	YES	YES	YES
	RAP1A	YES	YES	YES
	RAP1B	YES	YES	YES
	RAP2A	YES	YES	YES
	RAP2B	YES	YES	YES
	RAP2C	YES	YES	YES
	RASD1	YES	YES	YES
	RASD2	YES	YES	YES
	RASL10A	NO	YES	YES
	RASL10B	NO	YES	YES
	RASL11A	NO	YES	YES
	RASL11B	NO	YES	YES
	RASL12	NO	YES	YES
	REM1	NO	YES	YES
	REM2	NO	YES	YES
	RERG	YES	YES	YES
	RERGL	NO	YES	YES
	RHEB	YES	NO	YES
	RHEBL1	YES	NO	YES
	RIT1	YES	NO	YES
	RIT2	YES	NO	YES
	RRAD	NO	YES	YES
	RRAS	YES	YES	YES
	RRAS2	YES	YES	YES

**Table 2:** Selection of Ras superfamily member RasGEF and RasGAP regulators. Selection of family members based on domain predictions using the SMART or Pfam databases

	Gene name	RAS domain in SMART	RAS domain in Pfam
RasGEF family	KNDC1	YES	YES
	PLCE1	YES	YES
	RALGDS	YES	YES
	RALGPS1	YES	YES
	RALGPS2	YES	YES
	RAPGEF1	YES	YES
	RAPGEF2	YES	YES
	RAPGEF3	YES	YES
	RAPGEF4	YES	YES
	RAPGEF5	YES	YES
	RAPGEF6	YES	YES
	RAPGEFL1	YES	YES
	RASGEF1A	YES	YES
	RASGEF1B	YES	YES
	RASGEF1C	YES	YES
	RASGRF1	YES	YES
	RASGRF2	YES	YES
	RASGRP1	YES	YES
	RASGRP2	YES	YES
	RASGRP3	YES	YES
	RASGRP4	YES	YES
	RGL1	YES	YES
	RGL2	YES	YES
	RGL3	YES	YES
	RGL4	YES	YES
	SOS1	YES	YES
	SOS2	YES	YES
RasGAP family	DAB2IP	YES	YES
	GARNL3	NO	YES
	NF1	YES	YES
	PLXNB1	NO	YES
	RALGAPA1	NO	YES
	RALGAPA2	NO	YES
	RAP1GAP	NO	YES
	RAP1GAP2	NO	YES
	RASA1	YES	YES
	RASA2	NO	YES
	RASA3	NO	YES
	RASA4	NO	YES
	RASAL1	NO	YES
	RASAL2	YES	YES
	RASAL3	YES	YES
	SIPA1	NO	YES
	SIPA1L1	NO	YES
	SIPA1L2	NO	YES
	SIPA1L3	NO	YES
	SYNGAP1	YES	YES
	TSC2	NO	YES

### Gene expression values and sums

The data sets labeled as *mm*NT_02, *dm*NT_01, *hs*CT_02, *hs*CT_03, and *hs*CL_02 are based on microarray measurements. For these data sets, total RNA levels were utilized. The remaining data sets are based on RNA sequencing technology. The RPKM (*hs*NT_01, *hs*NT_03, *hs*NT_04, *hs*NT_05, *ggo*NT_01, *pt*NT_01, *pp*NT_01, *ppy*NT_01, *mmul*NT_01, *mm*NT_01, *md*NT_01, *oa*NT_01, *gg*NT_01, *hs*CT_01) or TPM (*hs*NT_02, *hs*CL_01) values (the way as presented by the original studies/databases) were utilized without applying any normalization or scaling factor. The sums for RAS, GEF, and GAP genes were computed by summing the original data for each corresponding gene. If there was more than one splice variant for a gene, the maximum expression variant was used. There are some genes missing in some of the data sets (especially in microarray-based sets). The missing gene expression level was assumed to be zero.

### Correlations

The gene expressions (array-based, RPKM or TPM) were used to determine the sum of GEF and GAP members, in different tissues/cell models with the Spearman’s rank correlation metric. Random correlations were determined based on independent shuffling of the expression levels of Ras family genes. The procedure was repeated 10,000 times, for each dataset. Finally, the real correlation value was compared to the distribution of shuffled correlations. The hypothesis that the real correlation value is significantly different than the random correlation distribution was tested via *p*-value, based on z-scores. To check that the high correlations were not dominated by one highly expressed family member, each GEF and GAP was removed one by one and the correlation was re-calculated (‘bootstrapping’). The effect of gene removal on ƩGEF to ƩGAP correlation values was not significant for either the normal tissue or the cancer tissue datasets. The significance of the difference between the matching normal and cancer dataset correlation values was determined by Fisher’s test; the correlation values were transformed to z-scores by using the sample size (number of individuals).

### Ratios

The gene expression levels from TCGA data set were used to determine the sum of GEF and GAP members, for the matching normal and cancer tissues. Mann-Whitney U test was applied to determine if the normal tissue ratios were significantly different from those of cancer tissues.

### Slope test

The gene expression values (array-based, RPKM, or TPM) were used to determine the sum of GEF and GAP members’ expression, for the matching normal and cancer tissues/ cell models. A non-parametric t-test (based on the regression line fitted to GEF GAP sums, the standard error of regression, the standard deviation of ∑GEF ∑GAP, and the number of individuals) was used to determine the significant changes between the slopes of the matching normal and cancer samples.

b_n_: slope of the fitted line for normal tissue.

b_c_: slope of the fitted line for cancer tissue.

Syx_n_: standard error of the fitted line for normal tissue.

Syx_c_: standard error of the fitted line for cancer tissue Sx_n_: standard deviation of the sum of GEF values in normal tissue.

Sx_c_: standard deviation of the sum of GEF values in cancer tissue

$$ {\displaystyle \begin{array}{l}{Sb}_n=\frac{Syx_n}{{Sx_n}^{\ast}\kern0.5em {\left({t}_n-1\right)}^2}\\ {}{Sb}_c=\frac{Syx_c}{{Sx_c}^{\ast}\kern0.5em {\left({t}_c-1\right)}^2}\end{array}} $$

where t_n_ and t_c_ are the number of individuals (data points) in normal and cancer tissue data sets, respectively.

$$ {\displaystyle \begin{array}{l}S={Sb}_n-{Sb}_c\\ {}T=\left({b}_n-{b}_c\right)/S\\ {} DF={t}_n-{t}_c-4\end{array}} $$

The corresponding *p*-value for the change in the slopes for normal versus cancer tissue is determined via t-test using the T and DF (degrees of freedom) values.

### Survival test

The TCGA data set was employed to determine the survival rates of patients with respect to their ∑RasGAP/∑RasGEF ratios. The “*TCGA2STAT*” R library was employed to download the matching RNA-Seq (RPKM) and clinical data sets. The clinical data were utilized to derive the “survival time” and “death/alive status” for each patient. The gene expression values were used to determine the sum of GEF and GAP members, and the corresponding ratio for each patient. For each cancer tissue, the corresponding normal tissue data set (TCGA) was used to determine ratio thresholds; “mean of normal ratio + 2 times standard deviation of normal ratio”. These tissue-specific ratio thresholds were used to stratify each patient as having a high- or a low-ratio. A survival function was fitted on the stratified data (based on high- or low-ratio), survival time, and death/alive status. The Kaplan-Meier plots for these fitted survival functions were depicted. The significance of difference between high- and low-ratio values are indicated on plots, with a p-value. The “*survminer*” R library was used for the survival analysis.

## Results

### Correlation of ∑RAS, ∑RASGEF and ∑RASGAP levels in adult normal tissues

We included in our study all RAS members and their GEF and GAP regulator members from human (*Homo sapiens*), other primates (*Gorilla gorilla*, *Macaca mulatta*, *Pan paniscus*, *Pan troglodytes*, and *Pongo pygmaeus*), other mammals (*Mus musculus*, *Monodelphis domestica*, and *Ornithorhynchus anatinus*), bird (*Gallus gallus*), fish (*Danio rerio*), and fly (*Drosophila melanogaster*), we used Pfam domain predictions and additional manual annotations based on recent reviews (Additional file 3) [12, 15].

We used public repositories and selected publications to retrieve gene expression data required for the subsequent workflow of performing correlations and ratios of RAS/ RASGEF/ RASGAP sums (Fig. 1c). The gene expression levels for RAS, RASGEF, and RASGAP members under different conditions (normal tissues and organs from various species, human cancer tissues, human cell lines) were retrieved and quantitative RNA Seq datasets were prioritised [28] (see Methods and Additional file 1: Figure S2). We analysed the gene expression correlations of the sums of RAS and regulators in adult fly (*dm*NT_01), chicken (*gg*NT_01), platypus (*oa*NT), opossum (*md*NT_01), mouse (*mm*NT_01, *mm*NT_02), primates (*mmu*NT_01, *ppy*NT_01, *pt*NT_01, *pp*NT_01, *ggo*NT_01), and human (*hs*NT_01, *hs*NT_02, *hs*NT_03, *hs*NT_04, *hs*NT_05) tissues (see Additional file 4). We found good correlations between the ∑GEF with the ∑GAP (Fig. 1d; Additional file 1: Figure S3A). Correlations between the ∑GEF with the ∑RAS and the ∑GAP with the ∑RAS were generally poorer (Fig. 1d). While a strong and significant (*p*-value < 0.05) correlation between the ƩGEFs and the ƩGAPs across tissues was found, the individual GEFs and GAPs showed a large distribution of correlation values (Additional file 1: Figure S3B), without any bias of higher correlations being associated to higher expression values (Additional file 1: Figure S3C). Thus, while the gene expression levels of individual GEF and GAP family members may vary in a given tissue, the sum of all GEFs and GAPs for a RAS subfamily is correlated.

To measure the significance of the correlation values, the RAS, GEF, and GAP gene expression values were shuffled 10,000 times and the correlation between the ƩGEFs and the ƩGAPs was determined (Additional file 5). We observed a strong correlation for the ƩGEF and the ƩGAP levels across normal adult tissues in most data in comparison to the shuffled background (*p*-value < 0.05) (Additional file 1: Figure S4). Focusing on a subset of experimentally validated GEFs and GAPs (RASGEF1A, RASGRP1, RASGRP4, SOS1, RASGRP2, NF1, RASA1, and RASA4) identified significant, but lower correlations values in all five human normal datasets (Additional file 5).

We also confirmed the ƩGEF to ƩGAP gene expression correlation across normal tissues at the protein level using the spectral counting score of the mass spectrometry-based proteomic meta-resource PaxDB [26]. The whole organism ∑RAS, ∑RASGEF and ∑RASGAP protein levels for human, mouse, worm, and fruit fly indicated no correlation between ∑RAS versus ∑RASGEF and also not between ∑RAS and ∑RASGAP. However, similar to the gene expression analysis, reasonable ∑RASGEF versus ∑RASGAP correlation values were observed for three organisms, but not for worm (Additional file 2). We further analysed the protein expression levels across human tissues (PaxDB [26] and Kim et al. [3]), where generally good correlation values between ∑RASGEF versus ∑RASGAP were found (Fig. 1e; Additional file 2). The correlation values of ∑RASGEF versus ∑RASGAP for protein and gene expression levels were in good agreement (Fig. 1f) even though not perfect – due to the low abundance of proteins. In conclusion, the overall correlation of ∑RASGEF and ∑RASGAP protein/gene levels in normal tissues indicates a balance of these entities.

### Correlation of ∑RAS, ∑RASGEF and ∑RASGAP levels in adult normal tissues across individuals

**Fig. 2:**
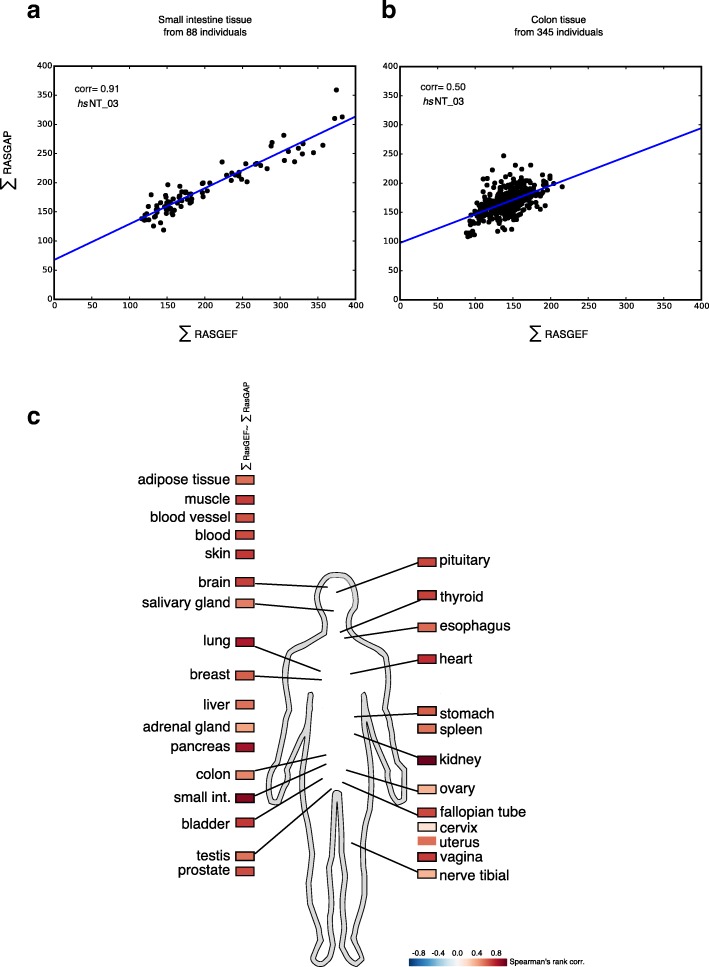
Spearman’s rank correlations in different tissues across individuals. **a** Correlation of the ∑GEF with the ∑GAP in small intestine across 88 individuals (GTEx, *hs*NT_03). **b** Correlation of the ∑GEF with the ∑GAP in colon across 149 individuals (GTEx, *hs*NT_03). **c** Heatmap of Spearman’rank correlation values across individuals for all tissue analysed. The part of the picture that represents a scheme of a human being was drawn using Inkscape

As the gene expression values from the human normal tissues from the GTEx consortium were average values of several individuals for each tissue (dataset *hs*NT_03), we next analysed whether or not the expression balance was even stronger in the same tissues of different individuals. In the small intestine tissue, for example, the ∑GEF and ∑GAP levels spanned a large expression range from 100 to 500 RPKM in different individuals, but the ∑GEF and ∑GAP levels were balanced in the same person (Fig. 2a). In other tissues that had a smaller dynamic range of expression (e.g. colon) we still found a significant correlation (Fig. 2b). We observed this expression balance throughout all/most tissues (Fig. 2c, Additional file 1: Figure S5, and Additional file 6). As observed before using the averaged tissue expression datasets, the correlation between RAS members and their GEFs or GAPs was generally lower (Additional file 6). In summary, the expression levels of GEF and GAP family members may vary in different individuals for a particular tissue, but the sum of all GEFs and GAPs was found to correlate in the same person.

### Gene expression ratios of RAS and regulators in normal adult tissues

**Fig. 3:**
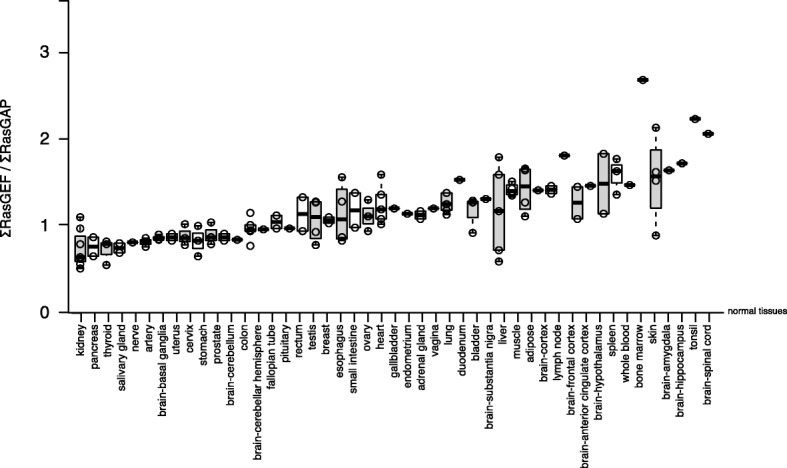
Ratios for the sum of RasGEF and RasGAP regulators in normal tissues. **a** Analysis of ∑RasGEF/∑RasGAP ratios in different human normal tissues (datasets *hs*NT_01, *hs*NT_02, *hs*NT_03, *hs*NT_04, and *hs*NT05). Similar tissues from different datasets were averaged (see Additional file 7)

To obtain insights into the quantitative relation among RAS and regulators, we compared the ratios of the ∑RAS, ∑GEF, and ∑GAP levels across the human normal tissues. We observed that the ratios for a given tissue were quite comparable in various datasets (Additional file 7; Additional file 1: Figure S6). We found that the average ∑GEF/∑GAP ratio across all tissues was 1.12, but ratios could be as low as 0.69 and as high as 2.57, suggesting that the ratio is constant in a particular tissue (Fig. 3). However, we could not identify a correlation between the ratio and tissue turnover times as a measure of tissue proliferation [29], suggesting that the ∑GEF/∑GAP ratio in individual tissues is unrelated to proliferation (Additional file 7).

### Loss of GEF and GAP expression correlation in human cancer tissues and cell lines

Correlation and clustering signatures previously revealed that various cancer types share a common gene expression profile, which differs from normal tissues and suggesting an underlying ‘near universal’ cellular dysfunction that is associated with cancer [30, 31]. Thus, we analysed the correlations between the ƩGEFs and ƩGAPs using cancer tissue expression datasets (datasets *hs*CT_01, *hs*CT_02, and *hs*CT_03) and cell lines (dataset *hs*CL_01 and hsCL_02) (Additional file 8). We found that the correlation value was often lower or lost in cancer tissues and cell lines (Additional file 1: Figure S7). Moreover, the correlation values for the cancer datasets were often not significantly different from the randomized data (Additional file 9). This suggests that some cancer tissues and cell lines do not have balanced ∑GEF and ∑GAP levels.

**Fig. 4:**
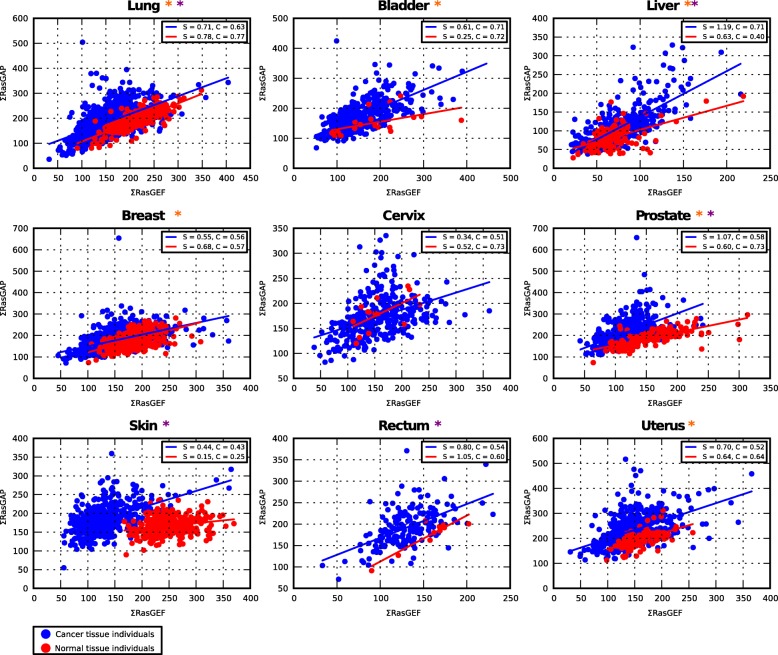
Correlations of the sum of RasGEF and RasGAP across individuals in different tissues. Spearman’s rank correlation of the ∑RasGEF with the ∑RasGAP in different normal (red squares) and cancer (blue squares) tissues across individuals (datasets *hs*NT_03 and *hs*CT_01). The correlation values (C) and slopes (S) are indicated in the figure. Significantly different changes in slopes or correlations comparing normal and cancer tissues are indicated next to the tissue name (plot titles), with orange and purple stars (*), respectively

To obtain further insights into the loss of correlation, we next analysed correlations across individuals in different cancer tissues (from the TCGA database). The TCGA data contain corresponding ‘normal’ tissue data that were taken from the same tissue, but from areas further away from the tumour (datasets *hs*CT_01 and *hs*NT_04). As the number of samples with from those ‘normal’ individual tissues were generally lower, we also analysed the normal tissues from individuals from the GTEx database (*hs*NT_03). The normal tissues from TCGA (*hs*NT_04) and GTEx (*hs*NT_03) generally agreed well when comparing the same tissue (Additional file 1: Figure S8; Additional file 10). For each tissue we performed correlations across individuals by treating the merged normal tissue data (*hs*NT_03 and *hs*NT_04) and cancer tissue data separately (Fig. 4; Additional file 1: Figure S8). Similarly to the overall correlations across cancer tissues, we observed that generally the correlations for specific cancer tissues were lower compared to normal tissues (Fig. 4; Additional file 1: Figure S8; Additional file 11) (16% of matching data and 23% of merged data had a significant change in correlation, *p*-value < 0.05). More importantly, however, we found that the slopes of ∑RASGEF-∑RASGAP were in five cases (bladder, liver, ovary, prostate, stomach) significantly increased, p-value 0.05 (Fig. 4; Additional file 1: Figure S8; Additional file 11). The increased slopes were due to increased ∑RASGAP levels in some tumours. However, there was a large heterogeneity in the tumours and only a fraction of tumours had higher ∑RASGAP levels. While there was no relation observed between the ∑GAP/∑GEF ratios and tumour stages (Additional file 12), for some cancer tissues (lung, cervix, and liver), a significant decrease in patient survival was observed for higher ∑GAP/∑GEF ratios suggesting that this ratio may be used as a prognostic indicator for survival (Additional file 1: Figure S9).

Taken together, these data suggest that the expression levels of small Ras-like GTPase regulators must be balanced to achieve the normal physiological functioning. The dysregulation of ∑GEF, ∑GAP and ∑RAS levels suggest that this may serve an additional hallmark of some cancers.

## Discussion

Here we show that sum of gene expression levels of GEF and GAP regulators of RAS GTPases is balanced in physiologically normal cell types and tissues. Genetic buffering and compensation has been shown to involve paralogs of genes that contain both functional overlap and functional differences [32]. The functional overlap for the families of GEFs or GAPs is given by their catalytic domains with GEF or GAP activity towards RAS small GTPases. The functional differences can be achieved using their multidomain nature containing a wide-range of different domains, which can affect their localization or specific functionalities such as the formation of complexes with other cellular proteins. Thus, individual activating (GEFs) and deactivating domains (GAPs) could change significantly their expression to perform particular functions using their other domains in particular tissues, but the overall balance for the GEF and GAP functionality is kept. The balanced expression of GEFs and GAPs should in principle enable a constant RAS activity (GTP vs GDP bound forms) in the normal tissues under constant growth signals, although protein levels do not necessarily relate 1:1 to protein activities. The finding that a subset of genes correlates weaker than the complete set but stronger than the random set is not unexpected. It is possible that, in future work, the identification of other subsets that correlate and characterizing their functions experimentally will further our understanding of the mechanisms of control of GEF/GAP expression and their role in the Ras pathway.

We also show that the GEF-GAP expression correlation is lost in some individual tumour tissues and cell lines. GEFs and GAPs have been frequently found to be differentially expressed in cancer [15, 33, 34]. Correlation and clustering signatures previously revealed that various cancer types share a common gene expression profile, which differs from normal tissues and suggests an underlying ‘near universal’ cellular dysfunction that results in cancer [30, 31]. Our work suggests that imbalances of GEF and GAP family members could be one feature of this dysregulation in some cancers. However, in is unclear if it is a cause or consequence of cancer development.

The fundamental mechanistic question of how levels of GEF and GAP are regulated and why they are altered in cancer tissues warrants further investigation. It is expected that the dosage of genes belonging to a functional group are under the control of signal transduction pathways and transcription factors [35]. For example, it has been shown that polo-like kinase 2 coordinates multiple GAPs and GEFs for synchronised tuning and activation of Ras and Rap small GTPases important for synaptic plasticity [36]. The most likely explanation being that the RAS activity state and downstream signalling itself induces positive and negative feedback mechanisms that control GEF and GAP levels through transcriptional regulation. Therein, a likely hypothesis is that levels are regulated by both GEF- and GAP-specific transcription factors and general transcription factors induced downstream of RAS.

It is quite remarkable that there are large differences in the sum of GEF and sum of GAP levels in the same tissue for different individuals, while in each individual a near perfect balance of sum of GEFs and GAPs is found. These individual expression differences are important to be considered in personalised medicine approaches, where for example a marker GEF protein that is highly expressed might be indicative of a bad prognosis in some individuals. However, in those individuals that have in general lower GEF and GAP levels this marker GEF protein might be missed if one compares to the average population expression levels.

Taken together, our data suggest that the expression levels of regulators of RAS GTPases must be balanced to achieve the normal physiological function in adult tissues. It is quite likely that similar phenomena are found in other multidomain groups of proteins sharing a functional domain. Thus, the finding that the protein paralogue expression levels quite often anticorrelate to keep constant a certain common functionality must now be extended to proteins sharing a common functional domain like GEFs and GAPs. For example, we have shown in mouse small intestine that knocking out the RhoGEF Tiam1 or Vav3 causes the upregulation of another RhoGEF, Vav2 (Pickering et al., manuscript under revision in Nature Communications). Only by knocking out all the three RhoGEFs simultaneously is suppression of the APC^fl/fl^ hyperproliferative phenotype observed.

## Conclusions

Our study shown that the function of multidomain proteins cannot be understood without considering all family homologs and explains why in many cases homologs exhibit expression anticorrelation. We suggest that future experimental analyses should include systemic changes of all protein family members rather than focusing on one particular protein. Further, our results are relevant for critical considerations of knock out experiments, where functionally related homologs may compensate for the down regulation of a protein.

## Additional files

Additional file 1: **Figure S1.** The Ras superfamily and regulators. **Figure S2.** Gene expression datasets analysed in this study. **Figure S3.** Example of sum and pairwise correlations. **Figure S4.** Correlations across adult normal tissues. **Figure S5.** Correlations across individuals in the background of random correlations. **Figure S6.** Example ratios in human lung tissues. **Figure S7.** Correlations in cancer tissues and cell lines. **Figure S8.** Correlations across individuals in normal and cancer tissues. **Figure S9.** Survival analysis of individuals with high and low RasGAP/RasGEF ratios. (DOCX 1465 kb)

Additional file 2: Protein expression levels and correlations in organisms and normal tissues. (XLSX 19 kb)

Additional file 3: RAS members and GEF and GAP regulators. (XLSX 83 kb)

Additional file 4: Ratios and correlations in human adult tissues. (XLSX 34 kb)

Additional file 5: Gene expression correlations for random shuffled genes in adult tissues. (XLSX 8257 kb)

Additional file 6: Gene expression correlations across human individuals in normal adult tissues. (XLSX 12 kb)

Additional file 7: Classification of human normal tissues into groups of similar tissues, ratio of sum of RASGEF to sum of RASGAP ratios, and tissue turnover times. (XLSX 13 kb)

Additional file 8: Ratios and correlations of gene expression levels in cancer tissues and cell lines. (XLSX 41 kb)

Additional file 9: Gene expression correlations for random shuffled genes in cancer tissues and cell lines. (XLSX 613 kb)

Additional file 10: Sum of RASGEF versus sum of RASGAP gene expression levels for human individuals in adult tissues and cancer tissues. (XLSX 296 kb)

Additional file 11: Slopes and correlations of sum of RASGEF versus sum of RASGAP gene expression levels in normal versus cancer tissues. (XLSX 14 kb)

Additional file 12: Gene expression correlations in cancer tissues according to tumor stages. (XLSX 11 kb)

## Abbreviations

COSMIC: Catalogue of somatic mutations in cancer
GAP: GTPase activating protein
GEF: Guanine nucleotide exchange factor
GTPase: Family of hydrolase enzymes that can bind and hydrolyse guanosine triphosphate (GTP)
RPKM: Reads per kilobase per million mapped reads
TCGA: The cancer genome atlas
TPM: Transcripts per kilobase million
