# Supplementary material for: Systems level expression correlation of Ras GTPase regulators
Source: Cell Commun Signal. 2018 Aug 15;16:46. doi: 10.1186/s12964-018-0256-8 (PMC6094892; doi:10.1186/s12964-018-0256-8)
Supplement: Supplementary file 1 — Figure S1. The Ras superfamily and regulators. Figure S2. Gene expression datasets analysed in this study. Figure S3. Example of sum and pairwise correlations. Figure S4. Correlations across adult normal tissues. Figure S5. Correlations across individuals in the background of random correlations. Figure S6. Example ratios in human lung tissues. Figure S7. Correlations in cancer tissues and cell lines. Figure S8. Correlations across individuals in normal and cancer tissues. Figure S9. Survival analysis of individuals with high and low RasGAP/RasGEF ratios. (DOCX 1465 kb) [file 12964_2018_256_MOESM1_ESM.docx]

Additional file 1


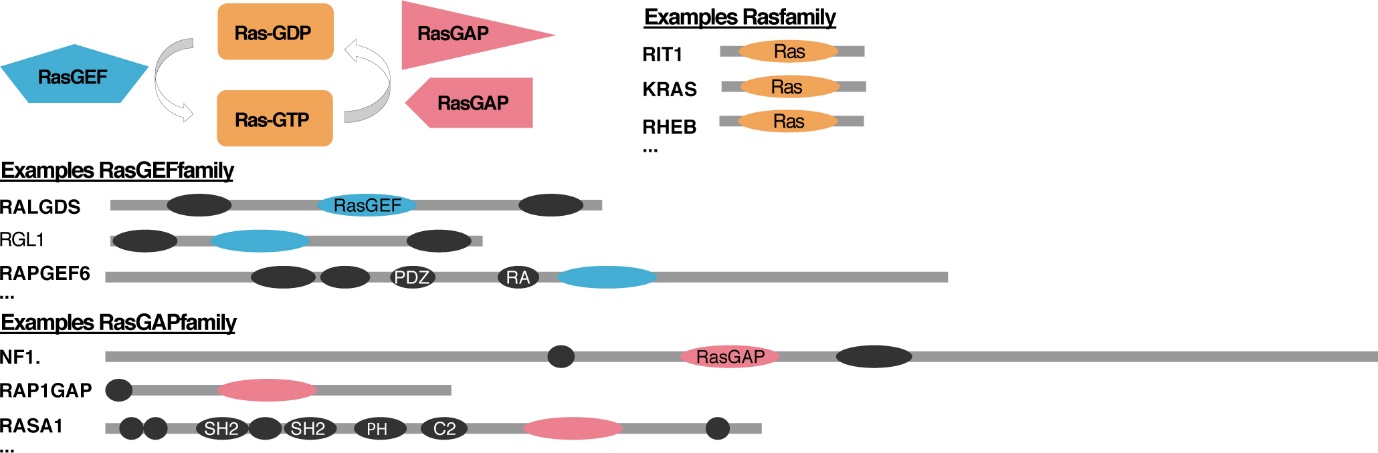


**Figure S1.** **Proteins and regulators of the Ras superfamily and examples of Pfam domain predictions.** The Ras subfamily with their GEF (containing the RasGEF domain) and GAP (containing RasGAP or RapGAP domains) proteins.

**
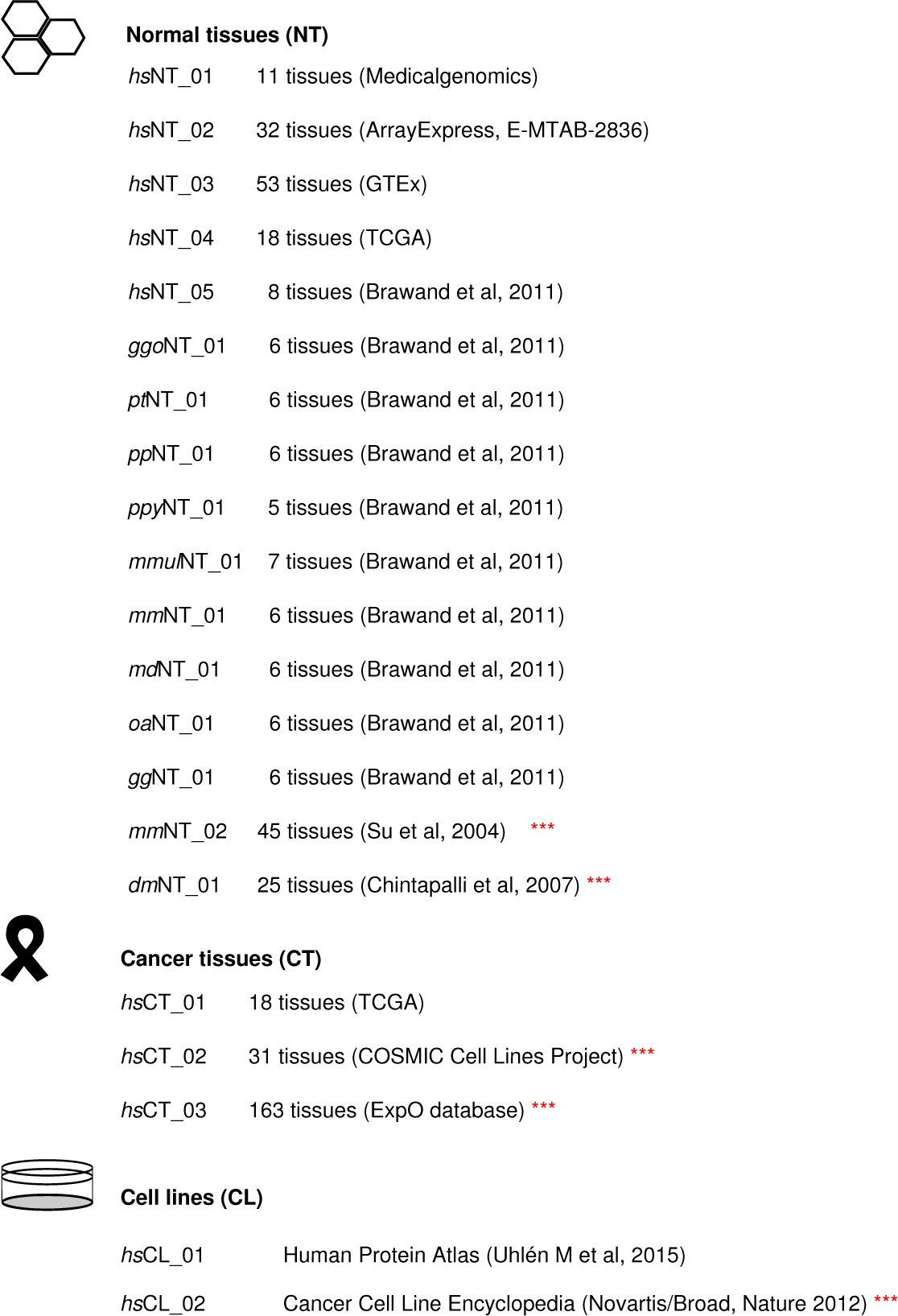
**

**Figure S2.** **Overview of gene expression datasets analysed in this study.** All datasets are from RNA sequencing, except the ones highlighted by red stars, which were obtained by array technologies. For details and references see the methods section. Parts of the pictures were drawn using Inkscape.

**
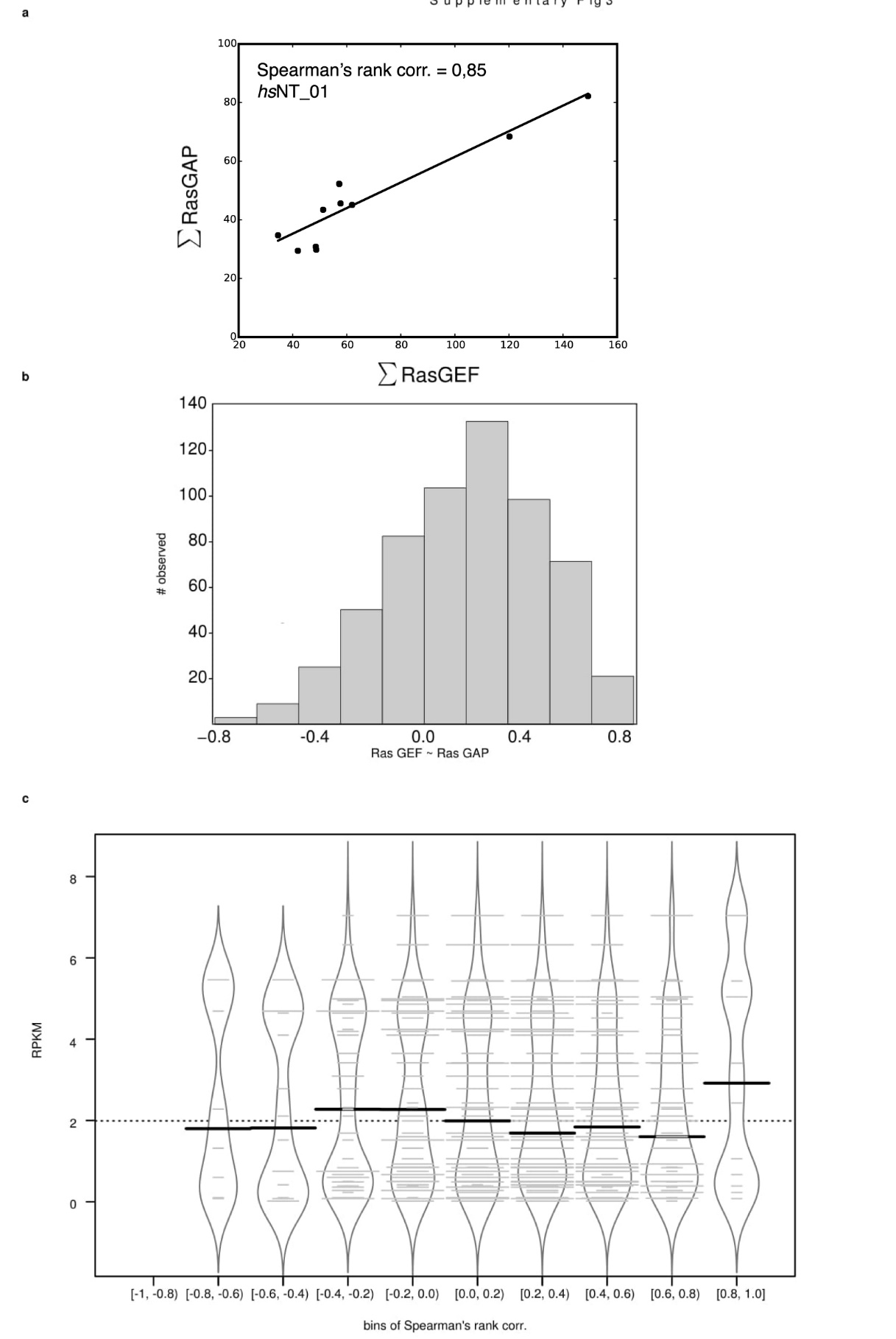
**

**Figure S3.** **Example correlation for the ΣGEF vs the ΣGAP for the Ras subfamily and pairwise correlations using the hsNT_01 dataset.** (a) Example correlation for the ΣGEF vs the ΣGAP for the Ras subfamily using the hsNT_01 dataset. (b) Example histogram of the pairwise correlations of individual Ras GEF and Ras GAP expression levels across 11 different tissues (dataset hsNT_01), where RPKM values of each gene were used. (c) Gene expression analysis for genes in the different bins of pairwise correlations as shown in panel b. The figure indicates the average expression (in different tissues) for each gene. The bins stand for the correlation values (each gene that contributes to that correlation is depicted as grey bar). The average expression for all genes in that bin is indicated by the black line.


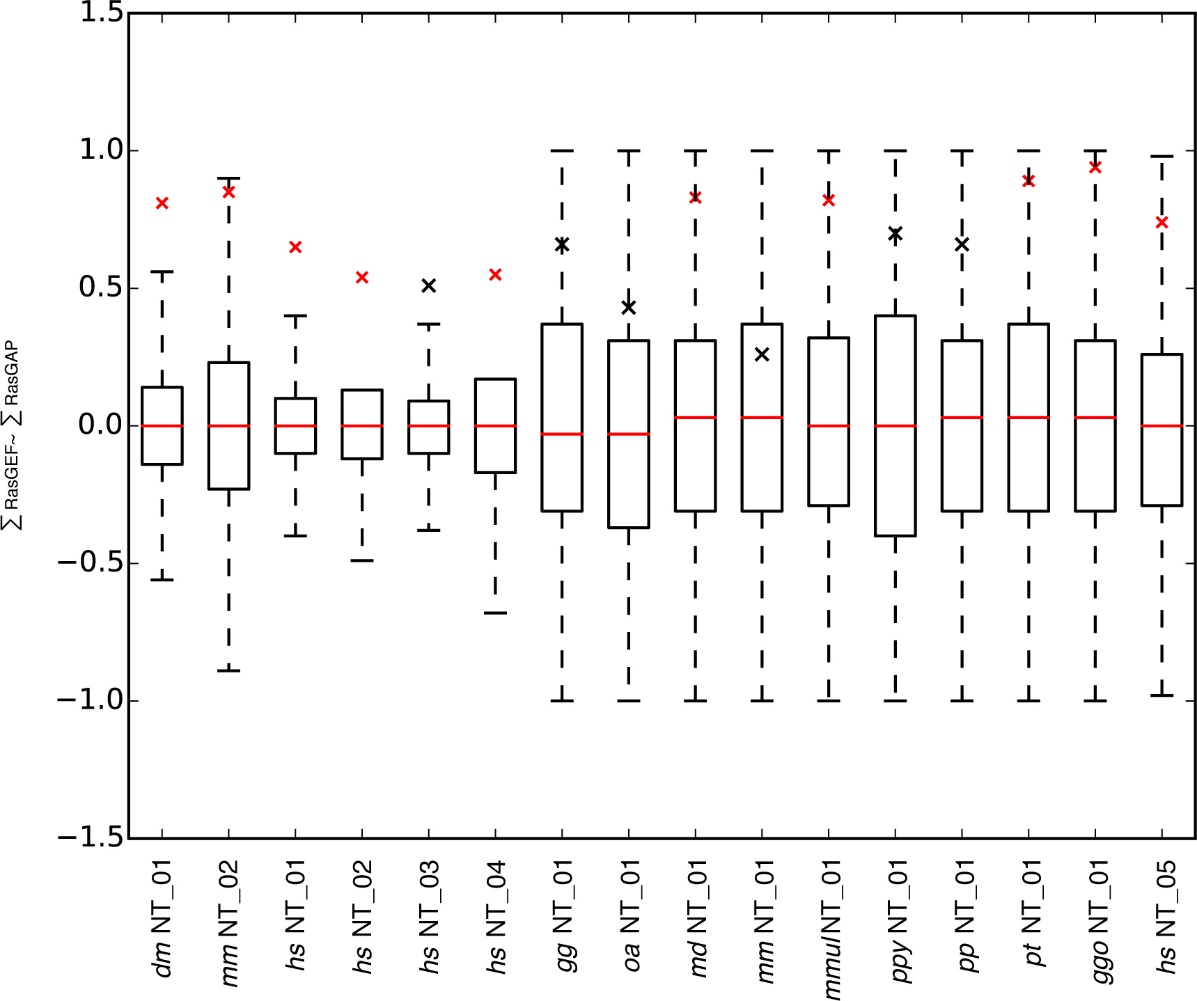


**Figure S4.** **Spearman’s rank correlations of the GEF with the GAP across adult normal tissues and organs in the background of random correlations.** Spearman’s rank correlations of the GEF with the GAP across adult normal tissues in the background of 10000 shuffled GEF vs GAP correlations for the Ras subfamily. The cross indicates the real correlation value and the boxplot/dots the random correlations. A red cross indicates that the real correlation is significant (p-value < 0.05) compared to the background of random correlations.


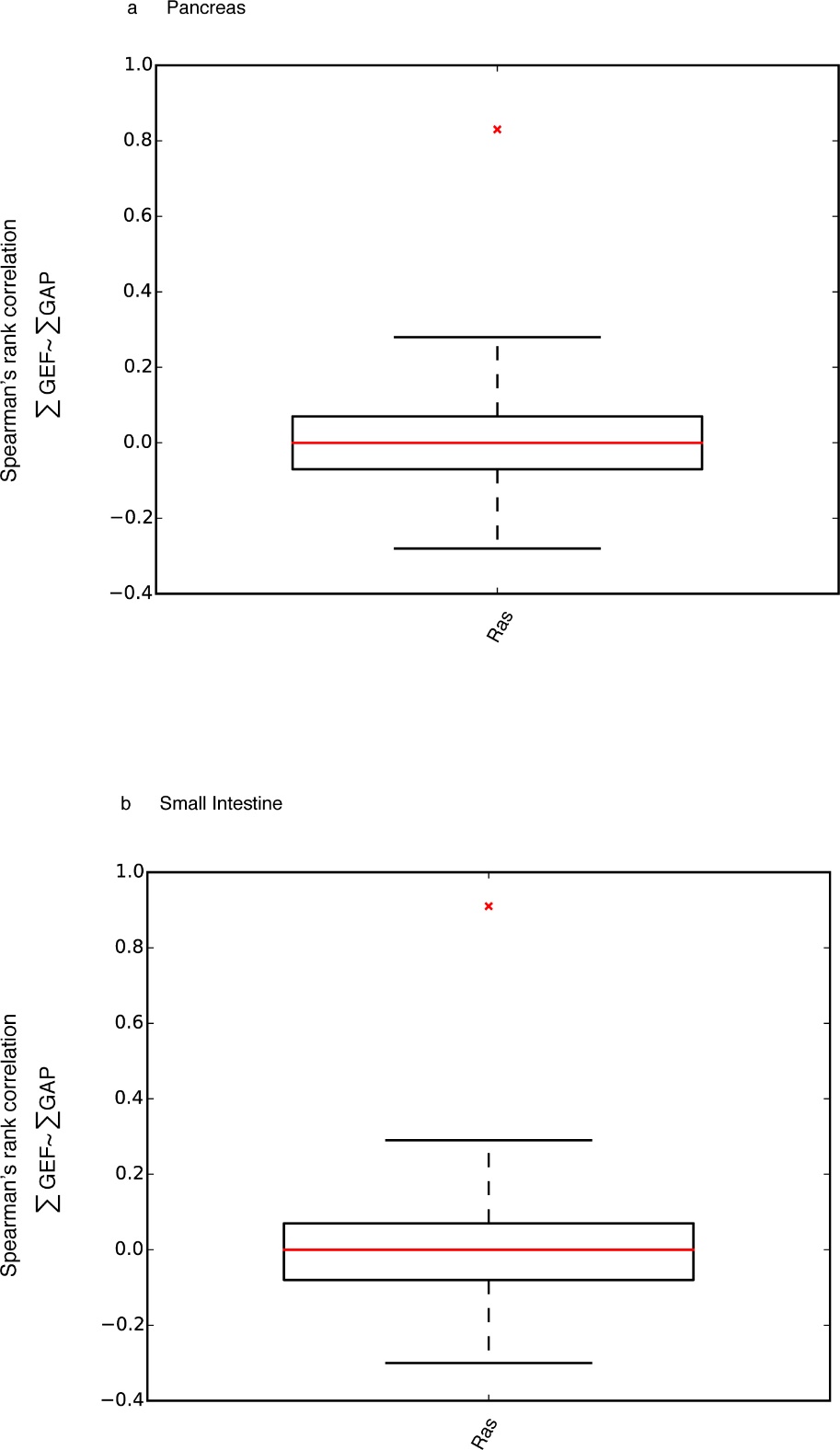


**Figure S5.** **Spearman’s rank correlations across individuals for pancreas and small intestine tissue in the background of random correlations.** Spearman’s rank correlations of the GEF GAP, across individual in the background of 10000 shuffled expression values for pancreas (panel a) and small intestine (panel b) tissues. The cross indicates the real correlation value and the boxplot/dots the random correlations. A red cross indicates that the real correlation is significant (p-value < 0.05) compared to the background of random correlations.


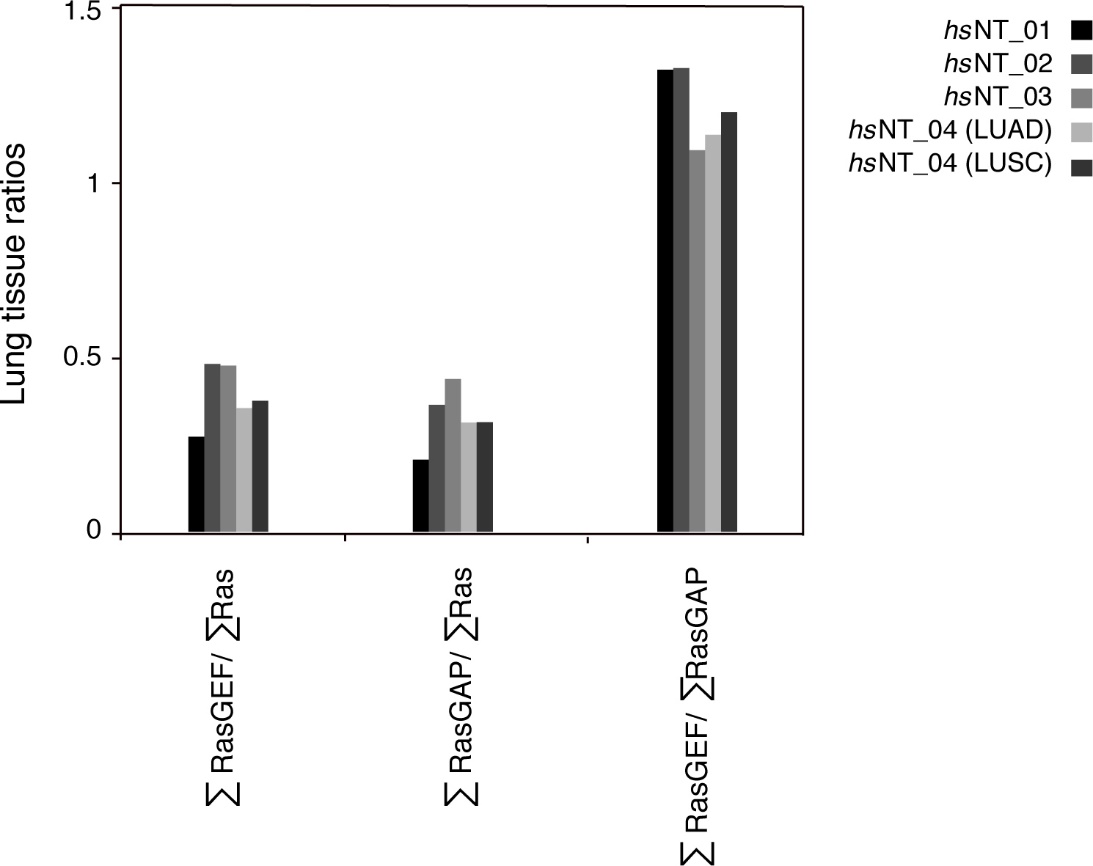


**Figure S6.** **Example ratios obtained from genes expression in human lung tissues.** Different ratios for the ΣRAS, ΣGEF, and ΣGAP for the four subfamilies are shown for the same tissue (lung) using different datasets (hsNT_01, hsNT_02, hsNT_03 and hsNT_04).


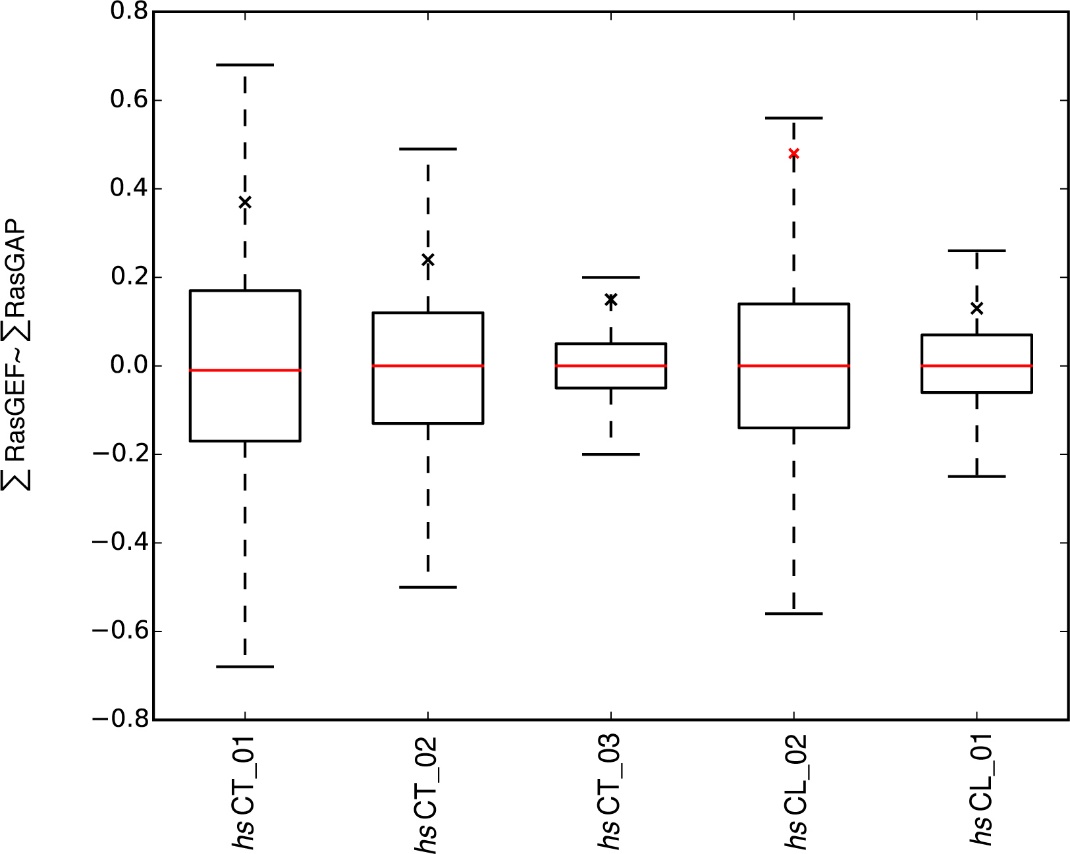


**Figure S7.** **Correlations of RAS and regulators in cancer tissues and cell lines.** Spearman’s rank correlations of the GEF with the GAP across cancer tissues and cell lines in the background of 10000 shuffled GEF vs GAP correlations. A red cross indicates that the real correlation is significant (p-value < 0.05) compared to the background of random correlations.


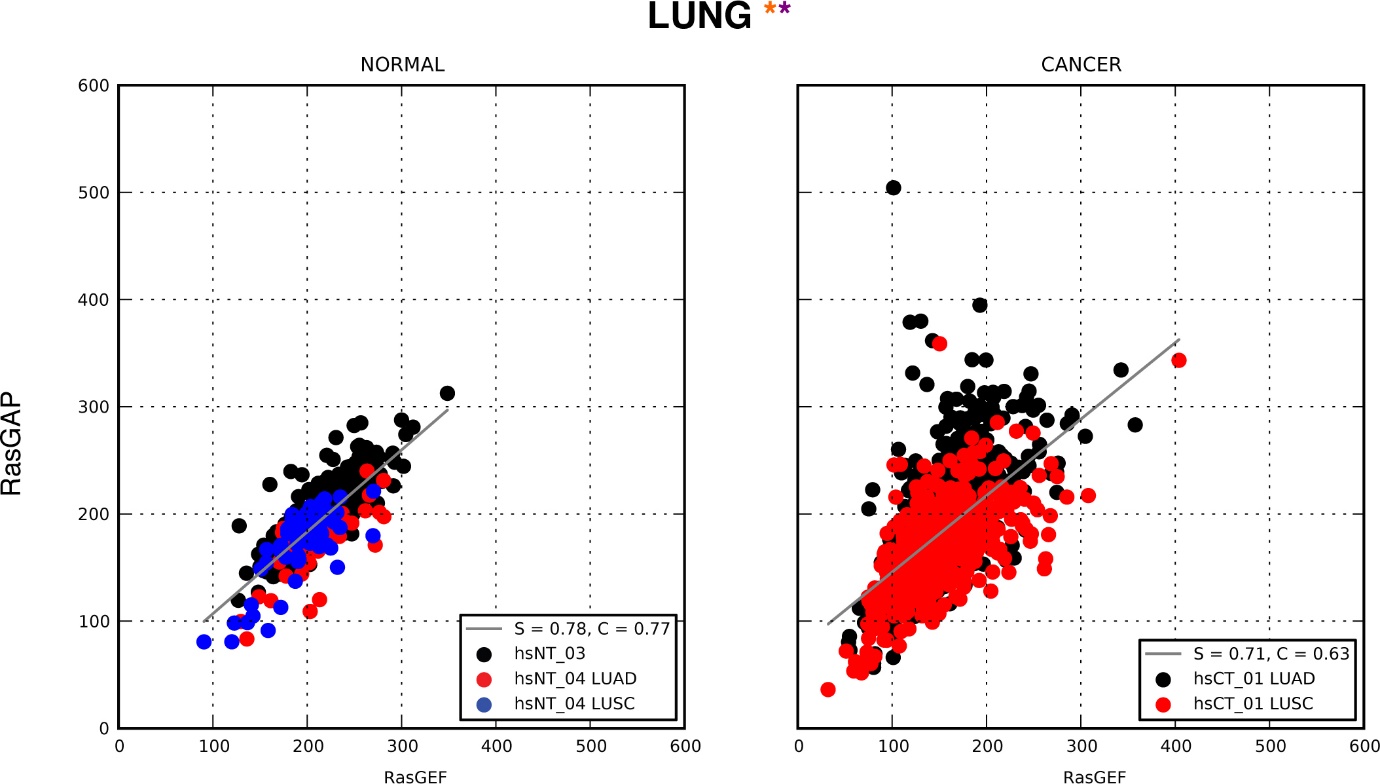


**Figure S8.** **Spearman's rank correlations for the sum of RasGEF and RasGAP regulators across individuals in normal and cancer tissues.** Correlation of the RasGEF with the RasGAP in different normal and cancer tissues across individuals (datasets hsNT_03, hsNT_04, and hsCT_01). Different colors of data points correspond to different (normal tissues) datasets. The regression line corresponds to all displayed datasets. The S and C letters in the legend correspond to the slope of the fitted line and the Spearman's rank correlation, respectively. A selection of summary plots is shown in main Figure4. Significantly different changes in slopes and correlations comparing normal and cancer tissues are indicated next to the tissue name (plot title), with orange and purple stars (*), respectively. There is no correlation between the sample size (number of individuals) and the dynamic range of sum of RasGEF and RasGAP levels.


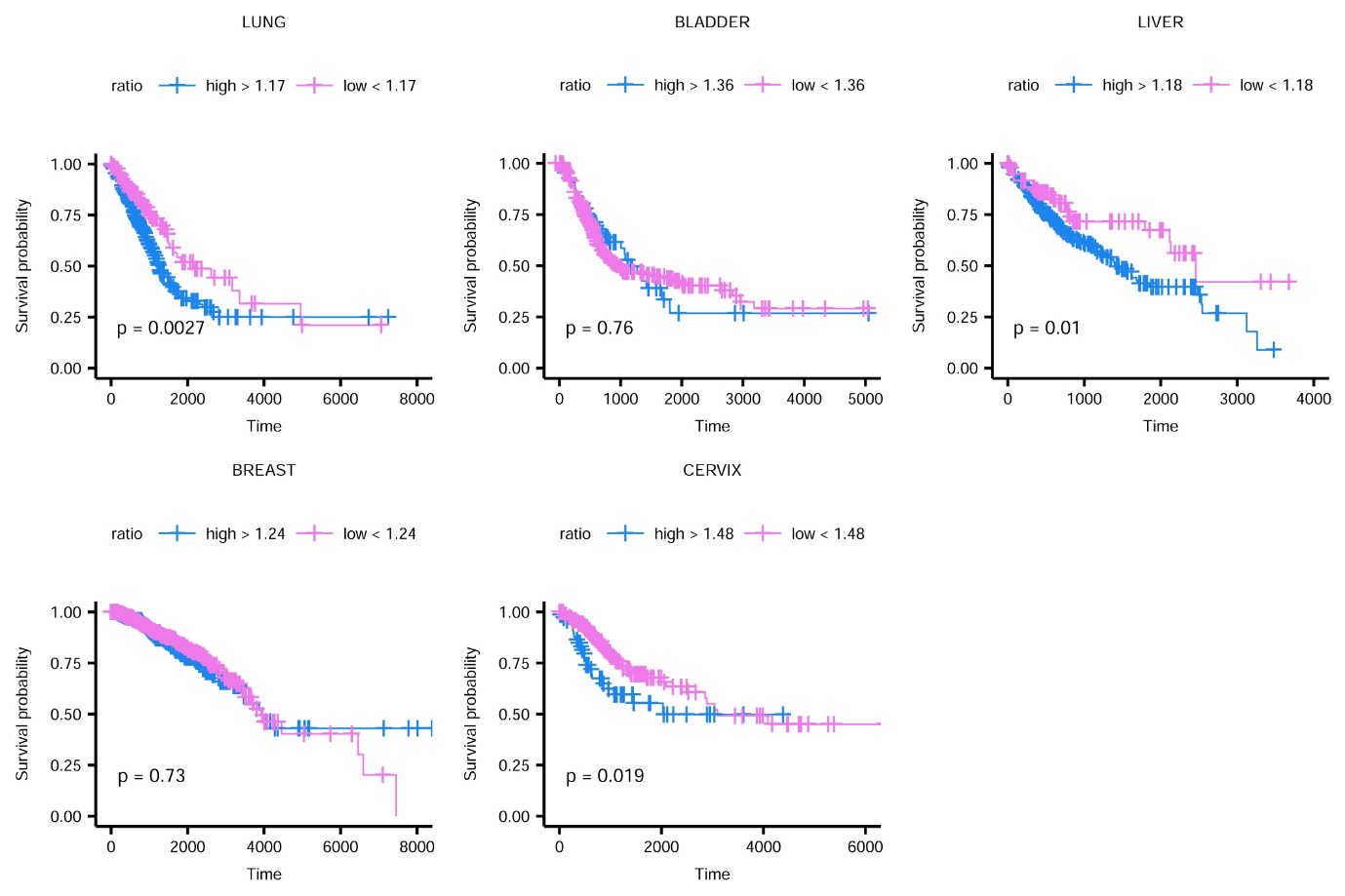


**Figure S9.** **Survival analysis of individuals with high (blue) and low (pink) RasGAP/RasGEF ratios given as Kaplan−Meier plots.** The p−value for each tissue is indicated on the corresponding plot.
